# Heterologous expression of pathogen-specific genes *ligA *and *ligB *in the saprophyte *Leptospira biflexa *confers enhanced adhesion to cultured cells and fibronectin

**DOI:** 10.1186/1471-2180-11-129

**Published:** 2011-06-09

**Authors:** Cláudio Pereira Figueira, Julio Croda, Henry A Choy, David A Haake, Mitermayer G Reis, Albert I Ko, Mathieu Picardeau

**Affiliations:** 1 Oswaldo Cruz Foundation, Brazilian Ministry of Health, Gonçalo Moniz Research Center, Rua Waldemar Falcão, 121, 40295-001 Salvador, Bahia, Brazil; 2 Veterans Affairs Greater Los Angeles Health Care System, Division of Infectious Diseases, 111F, 11301 Wilshire Blvd, Los Angeles, CA 90073, California, USA; 3 Department of Medicine, University of California Los Angeles School of Medicine, Los Angeles, California, USA; 4 Division of Epidemiology of Microbial Diseases, Department of Epidemiology and Public Health, Yale University School of Medicine, Division of Epidemiology of Microbial Disease, 319 LEPH - 60 College St., New Haven, CT 06510 New Haven, USA; 5 Institut Pasteur, Unité de Biologie des Spirochètes, 28 rue du docteur Roux, 75724 Paris Cedex 15, France; 6 Faculty of Health Sciences, Federal University of Grande Dourados, Brazil

## Abstract

**Background:**

In comparison to other bacterial pathogens, our knowledge of the molecular basis of the pathogenesis of leptospirosis is extremely limited. An improved understanding of leptospiral pathogenetic mechanisms requires reliable tools for functional genetic analysis. Leptospiral immunoglobulin-like (Lig) proteins are surface proteins found in pathogenic *Leptospira*, but not in saprophytes. Here, we describe a system for heterologous expression of the *Leptospira interrogans *genes *ligA *and *ligB *in the saprophyte *Leptospira biflexa *serovar Patoc.

**Results:**

The genes encoding LigA and LigB under the control of a constitutive spirochaetal promoter were inserted into the *L. biflexa *replicative plasmid. We were able to demonstrate expression and surface localization of LigA and LigB in *L. biflexa*. We found that the expression of the *lig *genes significantly enhanced the ability of transformed *L. biflexa *to adhere *in vitro *to extracellular matrix components and cultured cells, suggesting the involvement of Lig proteins in cell adhesion.

**Conclusions:**

This work reports a complete description of the system we have developed for heterologous expression of pathogen-specific proteins in the saprophytic *L. biflexa*. We show that expression of LigA and LigB proteins from the pathogen confers a virulence-associated phenotype on *L. biflexa*, namely adhesion to eukaryotic cells and fibronectin *in vitro*. This study indicates that *L. biflexa *can serve as a surrogate host to characterize the role of key virulence factors of the causative agent of leptospirosis.

## Background

The genus *Leptospira *belongs to the order *Spirochaetales *and includes both saprophytic and pathogenic members, such as *Leptospira biflexa *and *L. interrogans*, respectively. Leptospirosis is the most widespread zoonosis worldwide, with more than one million cases annually [1,2]. Rodents are the principle reservoir of infections occurring in humans, resulting from renal tubular colonization and urinary excretion of the bacterium [3]. Humans are usually infected through water that is contaminated with the urine of animal reservoirs. This increasingly common disease primarily occurs in rural environments and poor urban centres subject to frequent flooding. A major barrier to developing effective control of the disease has been our limited understanding of the biology of the bacterium. One of the reasons for this is the slow growth of pathogenic leptospires with a generation time of approximately 20 hours; colonies can take up to 4 weeks to appear on solid medium [4]. Furthermore, there are fewer tools for genetic studies of pathogenic leptospires than are available for many other bacterial pathogens. Tools for genetic manipulation of the saprophyte *L. biflexa *have been developed in recent years [4]. This work has significantly improved the feasibility of manipulating genes in pathogenic strains. For instance, we first developed systems for targeted mutagenesis and random transposon mutagenesis in the saprophyte *L. biflexa *and then applied these approaches in the pathogen *L. interrogans *[5-7]. However, the introduction of exogenous genetic information into pathogenic strains by electroporation [8] or conjugation [9] is still hindered by poor transformation efficiencies. In addition, there is no replicative plasmid vector available for pathogenic *Leptospira *strains. Further development and improvement of genetic tools is therefore necessary for functional analysis of leptospiral virulence factors.

High-molecular-weight leptospiral immunoglobulin-like repeat (Lig) proteins were previously identified as putative virulence factors in pathogenic *Leptospira *spp. [10-12]. This family of three proteins - LigA, LigB and LigC - belongs to the superfamily of bacterial immunoglobulin (Ig)-like (Big) repeat domain proteins which includes virulence determinants such as intimin from enteropathogenic *Escherichia coli *and invasin from *Yersinia pseudotuberculosis *[10]. This superfamily appears to mediate pathogen-host cell interactions, such as invasion and host cell attachment, during infection. Several studies recently showed that recombinant Lig proteins can mediate *in vitro *interaction with fibronectin, fibrinogen, collagen, laminin, tropoelastin, and elastin [13-15]. Fibronectin-binding sites have also been identified in LigB [14,16,17] and fibronectin-binding activity was shown to be modulated by calcium [18]. In addition, *lig *genes are up-regulated at physiological osmolarity [52] and encode surface-exposed proteins that are strongly recognized by sera from human leptospirosis patients [11,19,20]. Lig proteins are also protective antigens in animal models of leptospirosis [10,21-25]. Taken together, these data suggest that Lig proteins are major virulence factors and may contribute to the pathogen's ability to attach to host tissues during infection. However, additional research is essential to understanding how *lig *gene expression modifies this phenotype. We recently showed that the absence of LigB does not lead to a loss of virulence and colonization in the acutely- and chronically- infected animal models [6]. This may be due to functional redundancy of other surface-exposed proteins, including LigA, in the bacterium.

Despite the large evolutionary distance between the pathogenic and non-pathogenic species, we have shown that the *Leptospira *genus shares a core of approximately 2000 genes, including those encoding the relevant export pathways [26]. The saprophyte *L. biflexa *could therefore represent a good cloning host for the functional analysis of genes from poorly transformable pathogenic *Leptospira*.

In this study, we used the non pathogen *L. biflexa *serovar Patoc as a surrogate host to characterize the role of LigA and LigB in leptospiral interactions with eukaryotic cells and key host extracellular matrix proteins.

## Results

### Expression of LigA and LigB in *L. biflexa*

**Figure 1:**
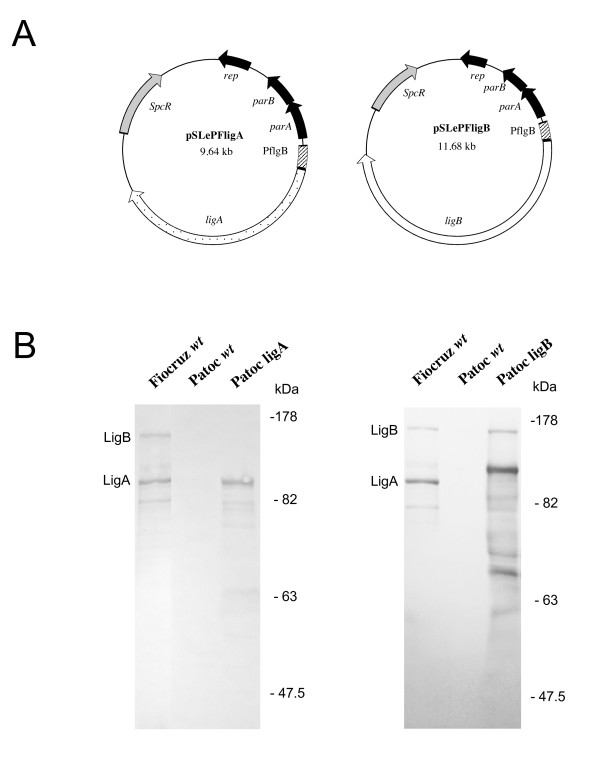
**LigA and LigB expression in *L. biflexa***. A. Schematic diagram of plasmid constructs used to express constitutively LigA and LigB. The determinants for replication in *L. biflexa *(*parAB *and *rep*), as well as a spectinomycin (SpcR)- resistance cassette is indicated. B. Western blot of whole-cell lysates of *L. interrogans *serovar Copenhageni strain Fiocruz L1-130 (Fiocruz wt), *L. biflexa *serovar Patoc strain Patoc 1 (Patoc wt), and *L. biflexa *serovar Patoc strain Patoc 1 electrotransformed with pSLEPFligA (Patoc ligA) and pSLEPFligB (Patoc ligB) obtained by using LigA/B antiserum. The positions of standard molecular mass markers (in kilodaltons) are indicated on the left.

The saprophyte *L. biflexa *can be transformed at high rates with plasmids based on the LE1 replication origin, using kanamycin, spectinomycin, or gentamicin resistance as the selectable marker [8,27,28]. We chose the spectinomycin-resistant plasmid, pSLe94, as the backbone for our system: this shuttle plasmid containing the LE1 partition genes is stably maintained in *L. biflexa *in the absence of antibiotic selection [27]. Flagellin-encoding genes are usually both constitutively and strongly expressed. In addition, it has been reported that a kanamycin resistant cassette driven by the *Borrelia burgdorferi flgB *promoter is strongly expressed in *B. burgdorferi *[29] and in *L. biflexa *[4]. We therefore used the *flgB *promoter from *B. burgdorferi *to allow strong and stable expression of LigA and LigB proteins in *L. biflexa *serovar Patoc (further indicated as Patoc). The genes encoding LigA and LigB under the control of the *flgB *promoter were inserted into the *L. biflexa *replicative plasmid (Figure 1A). The Patoc wild-type (wt) strain was then electrotransformed by pSLePF*ligA *and pSLePF*ligB*, and the spectinomycin-resistant transformants were further analyzed. Lig expression by the *lig*-transformed Patoc strains was verified by Western blot analysis, which showed levels of protein comparable to the production by a low *in vitro*-passaged *L. interrogans *virulent strain (i.e. less than 10 *in vitro *passages). However, blots of the *ligB *transformant showed partial degradation of LigB (Figure 1B). The Patoc wt, *ligA*, and *ligB *strains had similar cell growth kinetics in EMJH liquid medium, indicating that the expression of the heterologous proteins did not affect cell growth (data not shown).

### Surface localization of LigA and LigB in *L. biflexa*

**Figure 2:**
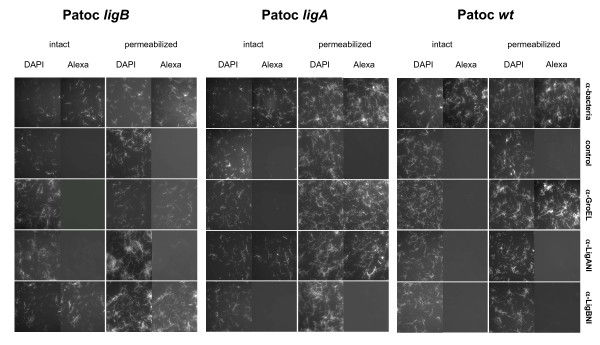
**Surface localization of LigA and LigB**. Surface immunofluorescence assay was performed with *L. biflexa *wild-type strain (Patoc wt), and *ligA*- (Patoc *ligA*), and *ligB*- (Patoc *ligB*) *L. biflexa *transformants. Strains were labeled with normal rabbit serum (control) and antibodies against LigA (LigANI), LigB (LigBNI), whole leptospires, and GroEL. A DAPI counterstain was used to document the presence of leptospires. A photomicrograph is shown from one of three representative experiments.

LigA and LigB proteins have been shown to be surface-exposed proteins in pathogenic *Leptospira *strains [11]. This was confirmed in this study with antibodies against LigA and LigB (see additional file 1: surface immunofluorescence assays in *L. interrogans*). Immunofluorescence studies found that antisera to LigA and LigB did not label the surface of the Patoc wt strain but did label the surface of the *ligA*- and *ligB*-transformed Patoc (Figure 2). The surface immunofluorescence binding assay specifically detected surface-exposed components because antiserum to whole bacteria labelled intact Patoc wt, Patoc *ligA*, and Patoc *ligB *whereas antisera to cytoplasmic heat-shock protein GroEL did not label live leptospires but was able to bind to permeabilized leptospires. LigA and LigB therefore appear to be surface-exposed when expressed in Patoc transformants carrying plasmid constructs pSLePF*ligA *and pSLePF*ligB*, respectively (Figure 2).

### Host cell adhesion and translocation of *lig*-transformed *L. biflexa*

**Figure 3:**
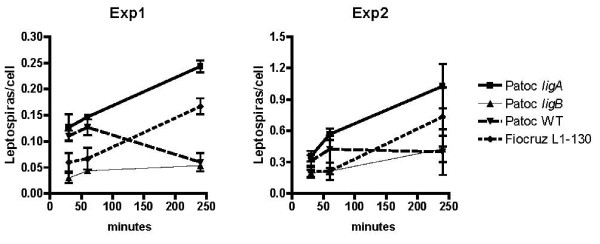
**Association of *L. biflexa *transformants with MDCK monolayers**. Adhesion of MDCK epithelial cells with *L. interrogans *(L1-130), *L. biflexa *wild-type strain (*wt*), and *ligA*- (*ligA*), and *ligB*- (*ligB*) *L. biflexa *transformants. Results were determined after 30, 60, and 240 minutes exposure, followed by extensive washing of non-adherent bacteria. The bars show the mean number of bacteria associated per host cell ± standard deviation carried out in 10 random fields in two independent experiments. The numbers of adherent leptospires/cell between the *L. biflexa *wild-type strain and the *ligA*- and *ligB*- *L. biflexa *transformants were statiscally different at 240 minutes (P < 0.05).

Interactions of Patoc wt, Patoc *ligA*, and Patoc *ligB *strains with mammalian host cells were assayed by examining adherence of leptospires to MDCK cells and translocation of leptospires across polarized MDCK cell monolayers. Adherence of *L. interrogans *strain Fiocruz L1-130 and Patoc *ligA*, but not Patoc wt and Patoc *ligB*, to MDCK cells was found to significantly increase in a time-dependent manner in two experiments (Figure 3). After a 240 min incubation period, approximately four times more Patoc *ligA *adhered to MDCK cells than Patoc wt and Patoc *ligB*. The number of adherent Patoc *ligA *leptospires per cell at 240 min incubation point was comparable (0.23 and 1.02 in experiments 1 and 2, respectively) to that observed for the pathogenic *L. interrogans *strain Fiocruz L1-130 (0.16 and 0.73 in experiments 1 and 2, respectively).

**Figure 4:**
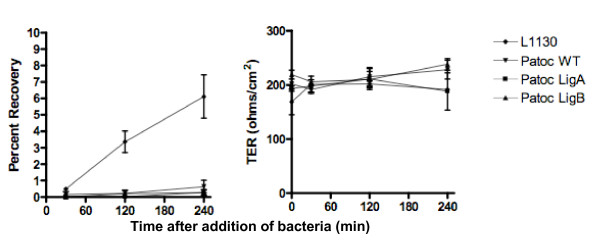
**Translocation assays**. Percent recovery of leptospires (left) and TER (right) after inoculation of polarized MDCK cell monolayers with *L. interrogans *Fiocruz L1-130 (L1-130), *L. biflexa *wild-type strain (Patoc wt), and *ligA*- (Patoc *ligA*), and *ligB*- (Patoc *ligB*) *L. biflexa *transformants. Bacteria were inoculated in the upper chamber of MDCK cell monolayer transwell chambers. Translocating bacteria was quantified by counting bacteria in the lower chamber. Assays were performed at 30, 120, and 240 minutes (min) after addition of bacteria. The assays were performed in triplicate, and results are expressed as mean ± SD. The findings of a representative experiment, among three which were performed, are shown.

Patoc *ligA *and *ligB *strains did not demonstrate enhanced ability to translocate across MDCK monolayers in comparison with Patoc wt in three experiments (representative experiment in Figure 4). As reported previously [30], we found that a small proportion (< 1%) of Patoc wt was able to translocate across MDCK monolayers after a 240 min incubation period. Proportions of translocating leptospires recovered from the lower transwell chamber were not significantly different between Patoc wt and Patoc *ligA *and *ligB *during the assay's time course (Figure 4). In contrast, > 6% of the inoculum of pathogenic *L. interrogans *strain Fiocruz L1-130 was recovered in the lower chamber after 240 min of incubation (Figure 4). As previously reported [30], recovery of *L. interrogans *strain Fiocruz L1-130 was not associated with significant alterations in the TER (Figure 4), indicating that disruption of tight junctions of the monolayers did not occur during the translocation process. Together these findings indicate that whereas expression of LigA in the saprophyte Patoc was associated with an enhanced host cell adherence phenotype similar to that observed with pathogenic leptospires, it did not impart the ability to efficiently invade and translocate across polarized host cell monolayers.

### Enhanced adhesion to fibronectin and laminin by *lig*-transformed *L. biflexa*

**Figure 5:**
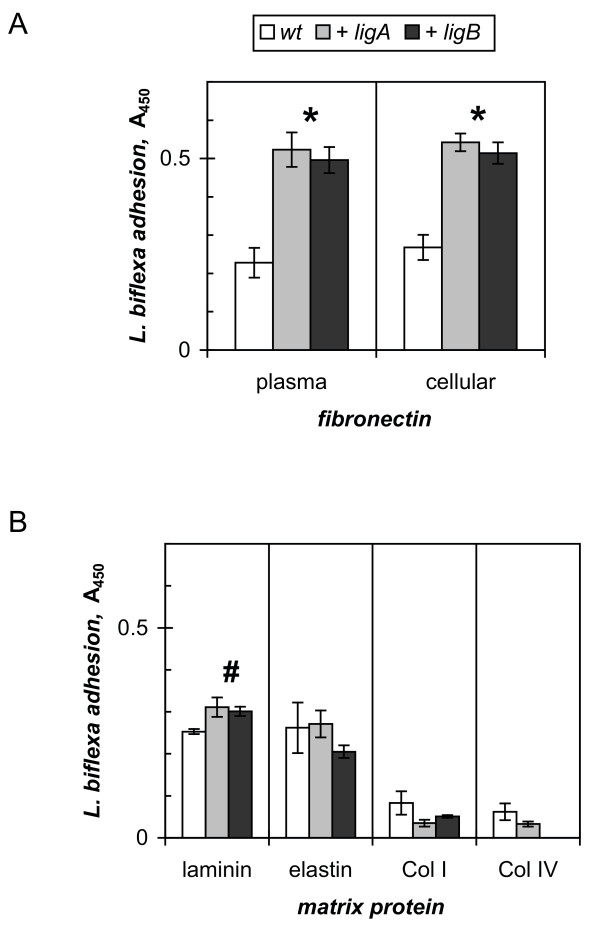
**Binding of *L. biflexa *transformants to extracellular matrix components**. A. Fibronectin binding assay was performed with *L. biflexa *wild-type strain (*wt*), and *ligA*- (+*ligA*), and *ligB*- (+*ligB*) transformed *L. biflexa*. The means and standard deviations of triplicates from a representative of more than three independent experiments are shown, with statistical significance at P < 0.01 (*). B. Laminin, elastin, and collagen type I (*Col I*) and type IV (*Col IV*) binding was measured as in A. with P < 0.05 (#).

Lig recombinant proteins have been shown to recognize *in vitro *host extracellular matrix proteins [13,14]. The introduction of the *ligA *or *ligB *gene from pathogenic *L. interrogans *into the nonpathogenic saprophyte *L. biflexa *enhanced the adhesion of the latter to the mammalian host protein fibronectin (Figure 5A). The *lig *transformants bound to both plasma and cellular fibronectin approximately two-fold better than the Patoc wild-type strain (2.0-fold average for 1.7- to 2.3-fold range in four independent determinations for the *ligA *cells; 2.2-fold average from 1.5- to 3.1-fold in five measurements with *ligB*). The wild-type cells showed non-Lig-mediated adherence to fibronectin, which may reflect the ability of the saprophyte to interact with related proteins in decaying material that it encounters in the environment. Transformation with the *lig *genes also increased laminin binding 1.2-fold in comparison to the Patoc wild-type strain (Figure 5B). However, the *ligA *or *ligB *cells did not appear to bind elastin better than wild-type cells, and all three strains interacted weakly with type I and type IV collagen (Figure 5B).

## Discussion

The lack of genetic tools has hampered molecular analyses of putative virulence factors in pathogenic *Leptospira *spp. In this work, we showed for the first time that pathogen-specific proteins can be expressed in a saprophytic *Leptospira *and that expression of such proteins are accompanied by an *in vitro *virulence associated phenotype. The approach used in this study demonstrates that the fast-growing non pathogenic species *L. biflexa *serves a model for examining pathogenetic mechanisms of *L. interrogans*. In contrast to *L. biflexa*, data obtained when *E. coli *was used as a surrogate host revealed that most of the spirochetal promoters functioned poorly in this genetic background. Even when leptospiral proteins are expressed in *E. coli*, many are found to be insoluble. An additional consideration is that a number of leptospiral proteins undergo post-translational modifications that may not occur in Gram negative bacteria [31].

In this study, the *L. interrogans *LigA and LigB lipoproteins were expressed and exposed on the surface of *L. biflexa *cells. However, the *ligB*-transformed *L. biflexa *produced almost no full length LigB protein. This suggests that *L. biflexa *is an appropriate surrogate host for expression of at least some *L. interrogans *outer membrane proteins [26]. These experimental results confirm genome sequence analyses indicating that most of the known protein export and processing systems of *L. interrogans *and *L. biflexa *are highly conserved [26]. Surface localization of Ligs in the model bacterium *L. biflexa *presents a unique opportunity to study the translocation of lipoproteins through leptospiral membranes. Further study could, for instance, include the analysis of the leptospiral lipobox which is distinct from the motifs of *E. coli *and other gram-negative bacteria. For example, the leptospiral surface lipoprotein, LipL41 was not efficiently expressed in *E. coli *until its lipobox was altered to mimic that of murein lipoprotein [32]. Analysis of leptospiral lipobox sequences indicates that most leptospiral lipoproteins would be anticipated to not be processed correctly in *E. coli *[33].

Bacterial adhesion is a crucial step in the infectious process.

Among members of the superfamily of bacterial immunoglobulin (Ig)-like (Big) proteins, previous studies have demonstrated that in comparison to the wild type strain, an intimin-deficient enteropathogenic *E. coli *strain is defective in adherence to cultured cells and in intestinal colonization [34]. In *Y. enterocolitica*, an invasin mutant was impaired in its ability to translocate the intestinal epithelium [35]. By contrast, we found that a *L. interrogans ligB^- ^*mutant retained its virulence and ability to adhere to MDCK cells [6]. This may be due to functional redundancy of other Lig proteins such as LigA. To determine the function of *lig *genes in pathogens, it may therefore be necessary to knock-out multiple genes, which would not be feasible in pathogenic *Leptospira *strains.

This study is a complete description of our approach for heterologous expression of pathogen-specific proteins in the saprophyte, *L. biflexa *serovar Patoc, resulting in the acquisition of virulence-associated phenotype. We demonstrate that Patoc *ligA *is able to adhere to epithelial cells in a time-dependent fashion, comparable to the pathogen *L. interrogans*. In addition, levels of binding of Patoc *ligA *and Patoc *ligB *to fibronectin and laminin were significantly higher in comparison to Patoc wt. However, *lig *transformants did not appear to bind collagens (type I and IV) or elastin better than wild-type cells. Analysis of Patoc *ligA *and Patoc *ligB *suggests that the Lig proteins are not sufficient for the efficient translocation of the bacteria across the cell monolayers, a characteristic that distinguishes leptospiral pathogens from saprophytes [30]. This result suggests that invasion is a more complex process than adherence and may require additional properties unique to leptospiral pathogens. In other words, invasion of cellular monolayers may require a stepwise adherence process involving interactions with a series of host ligands. Recently, we described enhanced fibrinogen binding of *L. biflexa *expressing LigA and LigB using the same plasmid constructs described here as part of a general examination of Lig-fibrinogen interactions [36], validating the relevance of our heterologous expression system.

Studies involving recombinant proteins, including LigA and LigB, have revealed a number of proteins that bind to extracellular matrix proteins [37-43]. Whether the functions of these putative adhesins are overlapping or synergistic in the interactions of leptospiral cells with eukaryotic cells or monolayers is unknown. LigA and LigB proteins contain related yet distinct Big domains that may share redundant function [13-15]. For example Choy *et al *demonstrated that portions of both LigA and LigB proteins bind fibronectin *in vitro *[13]. Thus the function of LigB can be substituted to varying extents by other lipoproteins, including LigA, which may play a role in host-cell interactions. The use of *L. biflexa *as a surrogate host enables functional studies of virulence factors in isolation without interference from activities of competing or redundant outer membrane proteins. Further studies expressing distinct regions of LigA and LigB in *L. biflexa *are required to understand the precise role of each domain in the binding of components of the extracellular matrix.

*L. interrogans *is an invasive pathogen that can adhere and translocate through host cells [30,44]. In contrast to the increased adherence of the *ligA*-transformed *L. biflexa *strain to MDCK renal cells, the *ligB *transformants did not exhibit enhanced attachment to the eukaryotic cells following four hours of incubation. This may be due to the partial degradation of LigB observed in these transformants by Western blots (Figure 1B). In contrast, we found that both *ligA*- and *ligB*-transformed *L. biflexa *bound fibronectin in significantly greater numbers than wild-type *L. biflexa *in a solid-phase assay format (Figure 5A). The large remaining LigB fragment appears slightly larger than intact LigA, suggesting that the degraded LigB may comprise the immunoglobulin-like repeats containing the fibronectin-binding domain [13]. These findings suggest that *lig*-mediated host cell adhesion may involve receptors in addition to fibronectin. The expression and localization of fibronectin and its binding integrins as well as other components of extracellular matrix in MDCK cells are dependent on culture conditions [45,46]. It is possible that the large proteolytic fragment of LigB remaining with the *ligB *transformants retains the fibronectin-binding region but has lost sequences mediating the interaction of LigB with a different and distinct renal cell receptor. Further studies with *lig *transformants could include analyzing *lig*-mediated host cell adhesion by using additional cell lines representing different species and cell types.

## Conclusion

In conclusion, by using *L. biflexa *as a surrogate host, we have shown that Lig proteins are factors involved in the attachment to fibronectin, fibrinogen, and laminin and to host cells and can act as microbial surface components recognizing host extracellular matrix proteins. Although important advances in the genetic system of the pathogen *L. interrogans *have been made in the last years [5,7], this bacterium remains poorly transformable and few mutants have been fully characterized [3]. We believe that *L. biflexa *can serve as a model bacterium for investigating the function of additional leptospiral pathogenesis mechanisms. Genetic studies in *L. biflexa *should provide information about the roles of key components in the pathogenesis of leptospirosis.

## Methods

### Bacterial strains and culture conditions

Leptospires were cultivated in liquid Ellinghausen-McCullough-Johnson-Harris (EMJH) medium [47,48] or on 1% agar plates at 30°C and counted in a Petroff-Hausser counting chamber (Fisher Scientific). The saprophyte *Leptospira biflexa *serovar Patoc strain Patoc I and the pathogen *L. interrogans *serovar Copenhageni strain Fiocruz L1-130 were used in this study. *E. coli *was grown in Luria-Bertani (LB) medium. When appropriate, spectinomycin or kanamycin was added to culture medium at the final concentration of 40 μg/ml.

### Plasmid constructions

The *Borrelia burgdorferi flgB *promoter was amplified with PflgA (5'-TAATACCCGAGCTTCAAGGAAG-3') and PflgB (5'-AACATATGGAAACCTCCCTC-3') and cloned into pCR2.1 (Invitrogen) to generate plasmid pCRPromFlgB. The *ligA *and *ligB *genes were amplified with flanking *Nde*I and *Xho*I sites, using primer pairs LANF (5'-GGGAATTCCATATGAAGAAAATATTTTGTATTTCG-3') - LAXR (5' CGGCTCGAGTTATTATGGCTCCGTTTTAATAGAGG-5') and LBNF (5'-GGGAATTCCATATGAAGAAAATATTTTGTATTTCG-5') - LBXR (5'-CGGCTCGAGTTATTATTGATTCTGTTGTCTGT-3'), respectively, from genomic DNA of *L. interrogans *serovar Copenhageni strain Fiocruz L1-130. Amplified *lig *genes were then digested with *Nde*I and *Xho*I restriction enzymes, purified, and inserted between the corresponding restriction sites of pCRPromFlgB to generate pCRP*flgB*LigA and pCRP*flgB*LigB, respectively. The DNA fragment containing Prom*_flgB _ligA (*4183 bp) and Prom*_flgB _ligB (*6188 bp) were released from plasmids pCRP*flgB*LigA and pCRP*flgB*LigB by *Spe*I and *Xba*I digestion, then blunt-ended, and cloned into the *Pvu*II restriction site of the *E. coli*-*L. biflexa *shuttle vector pSLe94 [49] to generate pSLePFligA and pSLePFligB (Figure 1). Plasmid constructs were verified by nucleotide sequencing.

### *lig*-transformed *L. biflexa*

*L. biflexa *was prepared for transformation as previously described [4]. In brief, *L. biflexa *was grown at 30°C until the optical density reached 0.4 at 420 nm. Bacteria were collected by centrifugation at room temperature and washed by resuspension in deionized water followed by centrifugation. After removing the supernatant fluid, the bacteria were resuspended with deionized water to a final concentration of around 5 × 10^10 ^cells/ml (100× concentration). 100 μl of the suspended bacteria were added to the plasmid DNA, and the DNA-bacteria mixture was added to chilled electroporation cuvettes with a 0.2 cm gap. The cuvette was placed in the electroporation unit (Bio-Rad Gene Pulser II) and subjected to electroporation at a setting of 1.8 kV, 25 μF, and 200 Ω. After adding 1 ml of EMJH, the bacteria were transferred to a 15 ml Falcon tube and incubated for 24 hours at 30°C with shaking. The culture (0.2 ml) was plated onto EMJH plates containing 40 μg/ml of spectinomycin and incubated at 30° for 10 days. Colonies were inoculated into liquid EMJH containing 40 μg/ml spectinomycin. *L. biflexa *transformants were maintained by serial passage in the liquid medium.

### Western Blot

Exponential phase cultures of *L. biflexa *Patoc wild-type, Patoc *ligA*, Patoc *ligB*, and *L. interrogans *Fiocruz strains were washed, resuspended in PBS and solubilized in 62.5 mM Tris hydrochloride (pH 6.8)-10% glycerol-5% 2-mercaptoethanol-2% sodium dodecyl sulfate. A 20 μl volume of crude extracts containing 2 × 10^8 ^bacteria/per well was resolved by 8% sodium dodecyl sulfate (SDS)-polyacrylamide gel electrophoresis using a discontinuous buffer system. After transfer to nitrocellulose membranes, immunoblots were blocked in 0.05 M Tris-buffered saline (pH 7.4)-0.05% (vol/vol) Tween 20 with 5% (wt/vol) nonfat dry milk. The blots were washed, incubated for 1 h at room temperature with a 1,000-fold dilution of mouse ascites containing MAb to the LigB identical repeat region (LigA/B) [6] and probed with goat anti-mouse conjugated to alkaline phosphatase (Sigma). Immunoblots were developed in a nitroblue tetrazolium--5-bromo-4-chloro-3-indolylphosphate (BCIP) solution (Bio-Rad).

### Localization of LigA/LigB by immunofluorescence

We evaluated the localization of LigA and LigB by performing immunofluorescence labeling according to a modified protocol of Cullen *et al*. [50]. Suspensions of 10^7 ^live leptospires in 10 μl of PBS were placed onto poly-L-lysine-coated slides (Sigma-Aldrich) for 1 h in a humidified chamber for adherence of the leptospires. In experiments in which the bacteria were permeabilized prior to incubation with antibody, slides were incubated with cold methanol for 10 min at -20°C, followed by two washes with PBS. Blocking with 1% bovine serum albumin (Sigma-Aldrich) (PBS-BSA) for 20 min was performed before incubation for 1 h at 37°C with normal rabbit serum, rabbit hyperimmune antisera to whole extracts of *L. interrogans *serovar Icterohaemorrhagiae strain RGA, LigB non-identical region (LigBNI), and LigA non-identical region (LigANI) [6], and rat antiserum to GroEL, which were diluted 1:100 in PBS-BSA. The slides were washed gently with PBS-BSA and incubated with goat anti-rabbit IgG antibodies conjugated to Alexa dye (Molecular Probes) or goat anti-rat IgG antibodies conjugated to fluorescein isothiocyanate (Jackson ImmunoResearch Laboratories) for 1 h at 37°C. The slides were washed twice with PBS-BSA and incubated with 1 μg/ml DAPI (Molecular Probes) for 1 h at room temperature. Slides were then washed, then mounted in anti-fading solution (Prolong-Molecular Probes) and visualized by fluorescence microscopy (Olympus BX51).

### Adhesion and translocation assays with MDCK cells

Madin Darby canine kidney (MDCK) cells were grown in Dulbecco's Modified Eagle's Medium (DMEM) supplemented with 10% fetal bovine serum (Cultilab), 2% sodium bicarbonate, 25 mM HEPES, and 5 mM L-glutamine (Sigma) at 37°C in an atmosphere of 5% CO_2_. MDCK cells were harvested by treating cell cultures with 0.05% trypsin and 0.02% EDTA in PBS. For adhesion assays, MDCK cells were plated onto 24-well plates in DMEM, containing 13-mm-diameter glass coverslips at 37°C in an atmosphere of 5% CO_2 _until they were confluent. The number of MDCK cells in wells was determined by lysing cells with 0.1 M citric acid containing 0.05% crystal violet (Sigma-Aldrich) and 1% Cetrimide (Sigma) [51], then the nuclei were counted in a hemacytometer. The cells were incubated with a suspension of Patoc wild-type, Patoc *ligA*, Patoc *ligB *and Fiocruz L1-130 strains in cell culture medium at the final bacteria: cell ratio of 10:1. Incubations were performed for periods of 30 to 240 min. Prior to staining, the cells were washed three times in PBS to remove nonadherent bacteria and then fixed with cold methanol for 10 min. An immunofluorescence assay was performed to detect adherent leptospires for which rabbit polyclonal antisera against whole extracts of *L. interrogans *strain RGA and goat anti-rabbit antibodies conjugated with Alexa488 (Molecular Probes) were used as first and second antibodies, respectively. DAPI and Alcian Blue were used to stain the nucleus and cytoplasm, respectively. The number of leptospires and MDCK cells was determined by examining ten high-magnification (1000×) fields during fluorescence microscopy. All incubation points were performed in triplicate. The ANOVA test was used to determine statistically significant (p < 0.05) differences between numbers of adherent leptospires/cell. We performed a translocation assay according to a protocol modified from that described by Barocchi et *al *[30]. MDCK cells at a concentration of 2 × 10^5 ^cells in 500 μl of DMEM were seeded onto 12-mm-diameter Transwell filter units with 3- μm pores. Monolayers were incubated at 37°C in 5% CO_2 _for 3 to 4 days with daily changes in media until the transmonolayer electrical resistance (TER) reached a range of 200 and 300 Ω/cm^2^, as measured with an epithelial voltohmmeter (EVOM, World Precision Instruments, Sarasota, Fla.). The TER for polycarbonate filters without cells was approximately 100 Ω/cm^2^. The upper chamber of the transwell apparatus was inoculated with leptospires at a multiplicity of infection (MOI) of 100 by adding 500 μL of bacteria which were resuspended in 1:2 v/v ratio of DMEM and EMJH media. Duplicate transwell chamber assays were performed for each leptospiral strain which were tested. Aliquots were removed from lower chamber (100 μl) at 30, 120 and 240 min and the number of leptospires was counted in triplicate by using the Petroff-Hausser chamber. The ability of leptospires to translocate MDCK polarized monolayers was determined by calculating the proportion of leptospires in the lower chamber in comparison to the initial inoculum for duplicate assays at each time point. The ANOVA test was used to determine significant differences in the proportions of translocating leptospires and TER values obtained during incubations with different leptospiral strains.

### ELISA for binding to extracellular matrix components

The adhesion of live *L. biflexa *strains to immobilized fibronectin was measured with an ELISA. Two to three × 10^8 ^cells in serum-free EMJH or medium alone was incubated at 30°C for 1 h in a microtiter well pre-coated with 1 μg of fibronectin (from human plasma or foreskin fibroblasts, Sigma-Aldrich), collagen type I (bovine skin, Sigma-Aldrich), collagen type IV (human placenta, Sigma-Aldrich), laminin (murine, Sigma-Aldrich), elastin (human skin, Elastin Products Company, Owensville, MO), or left in PBS, pH7.2, overnight at 4°C. Uncoated sites in the well were covered with Protein-Free Blocker (Thermo Scientific) before the addition of cells. Adherent cells were fixed with 4% formaldehyde (Thermo Scientific) at room temperature for 1 h, tagged with a rabbit polyclonal antibody for intact *L. biflexa *(MyBioSource), and detected by spectrometry at 450 nm to measure the activity of horseradish peroxidase conjugated to a donkey antibody for rabbit IgG (GE Healthcare). Backgrounds from uncoated wells (PBS) and medium only were subtracted. Triplicate assays were done and statistically significant differences in adhesion were determined with one-way ANOVA compared to the wild-type cells.

## Authors' contributions

AIK, DAH, HAC, and MP conceived the study. JC generated the plasmid constructs. CPF performed immunofluorescence, adhesion, and translocation assays. HAC performed the fibronectin binding assays. CPF, AIK, DAH, HAC, MGR, and MP participated in data interpretation and manuscript preparation. All authors read and approved the manuscript.

## Supplementary Material

Additional file 1 **surface immunofluorescence assays in *L. interrogans***. Immunofluorescence assays were performed with *L. interrogans *strain Fiocruz L1-130, which was labeled with normal rabbit serum (control) and antibodies against LigA (LigANI), LigB (LigBNI), GroEL, and LPS. Alexa- and fluorescein isothiocyanate-conjugated secondary antibodies were used to detect surface-bound antibodies. A DAPI counterstain was used to document the presence of leptospires. The photomicrograph show the results of one of three representative experiments. Click here for file
