# Supplementary figures and images for: Heterologous expression of pathogen-specific genes ligA and ligB in the saprophyte Leptospira biflexa confers enhanced adhesion to cultured cells and fibronectin
Source: BMC Microbiol. 2011 Jun 9;11:129. doi: 10.1186/1471-2180-11-129 (PMC3133549; doi:10.1186/1471-2180-11-129)

## Slide 1
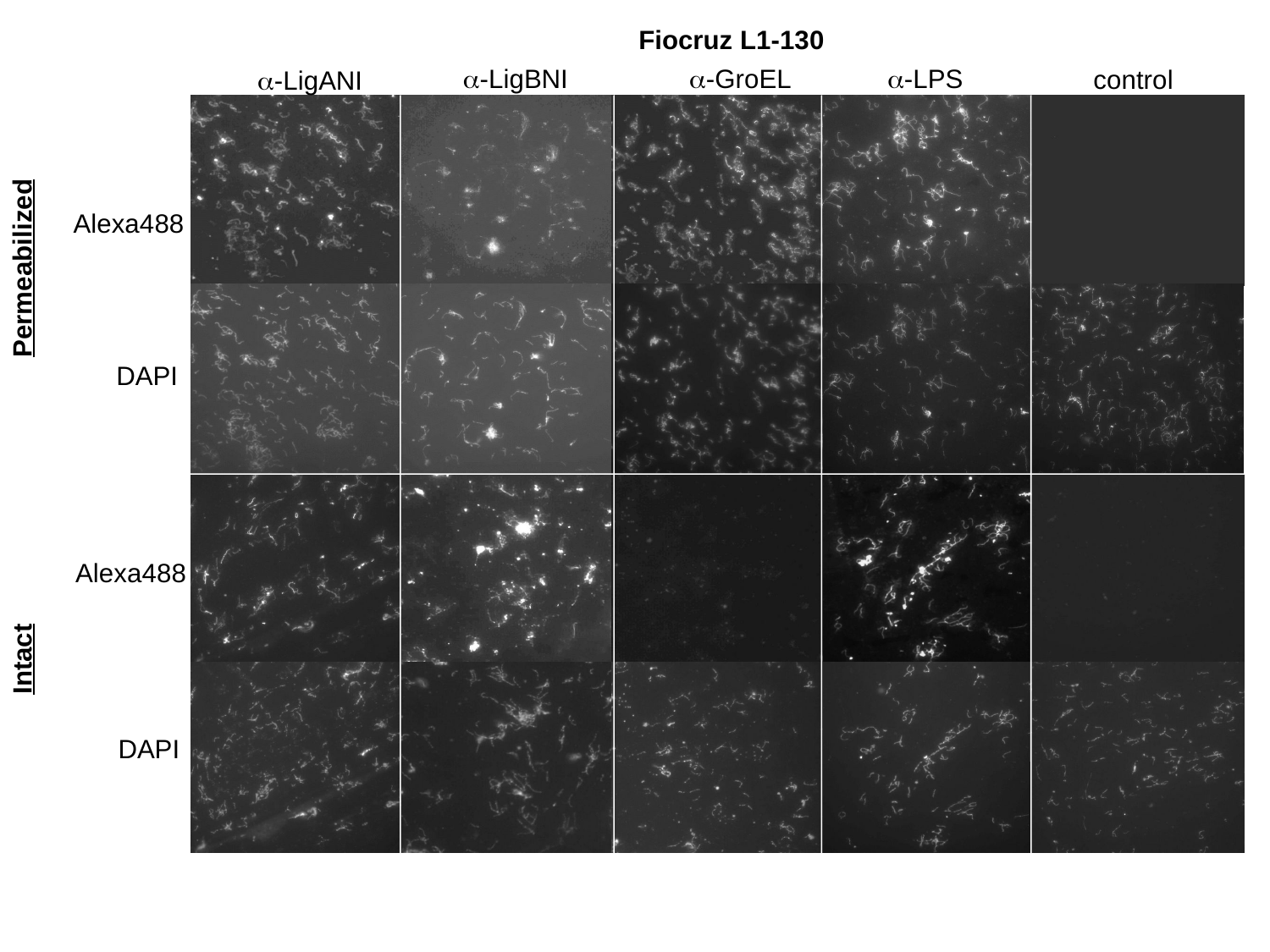

Fiocruz L1-130
α-LigBNI
α-GroEL
α-LPS
control
α-LigANI
Alexa488
Permeabilized
DAPI
Alexa488
Intact
DAPI

Supplement: Additional file 1 — surface immunofluorescence assays in L. interrogans. Immunofluorescence assays were performed with L. interrogans strain Fiocruz L1-130, which was labeled with normal rabbit serum (control) and antibodies against LigA (LigANI), LigB (LigBNI), GroEL, and LPS. Alexa- and fluorescein isothiocyanate-conjugated secondary antibodies were used to detect surface-bound antibodies. A DAPI counterstain was used to document the presence of leptospires. The photomicrograph show the results of one of three representative experiments. [file 1471-2180-11-129-S1.PPT]
